# Effect of* Betula pendula* Leaf Extract on *α*-Glucosidase and Glutathione Level in Glucose-Induced Oxidative Stress

**DOI:** 10.1155/2016/8429398

**Published:** 2016-09-07

**Authors:** Kristina Bljajić, Nina Šoštarić, Roberta Petlevski, Lovorka Vujić, Andrea Brajković, Barbara Fumić, Isabel Saraiva de Carvalho, Marijana Zovko Končić

**Affiliations:** ^1^Faculty of Pharmacy and Biochemistry, University of Zagreb, A. Kovačića 1, 10 000 Zagreb, Croatia; ^2^Faculty of Sciences and Technology, University of Algarve, Campus de Gambelas, Bd. 8, 8005-139 Faro, Portugal

## Abstract

*B. pendula* leaf is a common ingredient in traditional herbal combinations for treatment of diabetes in southeastern Europe. Present study investigated* B. pendula* ethanolic and aqueous extract as inhibitors of carbohydrate hydrolyzing enzymes, as well as their ability to restore glutathione concentration in Hep G2 cells subjected to glucose-induced oxidative stress. Phytochemical analysis revealed presence of rutin and other quercetin derivatives, as well as chlorogenic acid. In general, ethanolic extract was richer in phenolic substances than the aqueous extract. Furthermore, a comprehensive analysis of antioxidant activity of two extracts (determined by DPPH and ABTS radical scavenging activity, total antioxidant activity, and chelating activity as well as ferric-reducing antioxidant power) has shown that ethanolic extract was better radical scavenger and metal ion reductant. In addition, ethanolic extract effectively increased cellular glutathione levels caused by hyperglycemia and inhibited *α*-glucosidase with the activity comparable to that of acarbose. Therefore,* in vitro* research using* B. pendula* plant extracts has confirmed their antidiabetic properties.

## 1. Introduction

Chronic hyperglycemia, which may arise as a consequence of diabetes or metabolic syndrome, induces oxidative stress in sensitive tissues because glucose in high concentrations forms reactive oxygen species (ROS). Elevated glucose concentrations, and thus induced oxidative damage, may adversely affect pancreatic islet *β* cells, leading to disturbances in insulin production and further aggravating hyperglycemic status. The harm caused by ROS causes damage and impairment of function of the classical secondary targets of diabetes, such as blood vessels, kidneys, nerves, and eyes [1], leading to cardiovascular diseases, as well as microvascular diabetic complications, including nephropathy, retinopathy, and neuropathy [2]. In addition to that, recent research has provided evidence that insulin resistance and impaired insulin signaling, typical for type 2 diabetes, may be a contributory factor to the progression of dementia and other neurological disorders [3].

Endo- or exogenous antioxidants play an important role in alleviating oxidative stress and its consequences. One of the most important cellular nonprotein antioxidants is glutathione (GSH). GSH protects cells by scavenging oxygen and nitrogen radicals and by reducing H_2_O_2_. GSH is also important in maintaining the concentration of other nonenzymatic antioxidants. For example, the oxidized vitamin C can be restored to the reduced form by enzymatic reaction which uses GSH as substrate [4]. Besides contributing to protection of cells against oxidative damage, GSH detoxifies xenobiotics and regulates the functions of redox-sensitive proteins [5]. However, in states of increased oxidants, production levels of glutathione in cells can deplete. Therefore, in such conditions, constant and rapid replenishment of GSH is required, which is accomplished through both the reduction of oxidized GSH and its* de novo* synthesis. However, high concentration of glucose leads to glycation of glutamate-cysteine ligase, the first enzyme of the glutathione biosynthetic pathway, thus leading to further decrease of GSH levels [6]. There is* in vitro* and clinical evidence that abnormally low levels of glutathione in cells may lead to *β*-cell dysfunction and the pathogenesis of long-term complications of diabetes. As a consequence, interest has been developed in the potential for therapeutic modification of glutathione status in the treatment of diabetes. For example, changing the GSH status can be achieved by using natural antioxidants such as lipoic acid [7], curcumin, or sulforaphane [4]. Such approach could be used for development of nutraceuticals with potential in the treatment of metabolic disorder and diabetes [6].

Besides their influences on GSH content, natural substances can exert other biological activities which can be beneficial in treatment of diabetes and its complications. For example, due to their antioxidant activity, directly or via their influence on endogenous antioxidants, they can protect cellular targets and consequently the tissues which are most susceptible to diabetic complications [8]. Besides that, they can influence the enzymes which participate in carbohydrate metabolism, such as *α*-amylase and *α*-glucosidase, thus retarding the postprandial increase of glucose concentration. Alpha-amylase is an enzyme secreted in saliva and pancreatic juice which catalyzes the hydrolysis of starch to a mixture of smaller oligosaccharides, which are then degraded to glucose by *α*-glucosidase, enzyme located in the mucosal brush border of the small intestine. Alpha-amylase and *α*-glucosidase inhibitors can thus be helpful in the development of compounds for the treatment of diabetes, obesity, and hyperlipemia. Medicinal plants may constitute a good source of *α*-amylase and *α*-glucosidase inhibitors [9, 10].

*Betula pendula*, Roth (Betulaceae) commonly known as silver birch, is a tree native to Europe and Asia. In traditional medicine of Eastern Europe, it is used as diuretic, especially in cases of cystitis, as well as treatment for rheumatism and arthritic diseases. Recent studies have shown that its diuretic potential may stem from its endopeptidases-inhibiting properties, while xanthine oxidase inhibitory properties may be responsible for its use in gout treatment [11, 12]. Furthermore,* B. pendula* leaf extract can inhibit growth and cell division of inflammatory lymphocytes [12], as well as inhibiting tyrosinase, the enzyme that catalyzes the first stages of melanin biosynthesis. Birch leaf extract also displayed antioxidant and metal chelating properties [13].

Besides its well known use as diuretic and anti-inflammatory drug,* B. pendula* leaf is often used as part of herbal mixtures for treatment of diabetes in traditional medicine of southeastern Europe. The aim of this work was to evaluate suitability of such use by studying the inhibitory effects of* B. pendula* extracts against *α*-amylase and *α*-glucosidase, as well as to evaluate their antioxidant and GSH protecting activities in hyperglycemic conditions. An additional aim was to determine the best solvent for extraction of active principles of* B. pendula* leaf. To the best of our knowledge, this is the first time that potential use of birch extract as supplementary treatment for diabetes has been investigated.

## 2. Materials and Methods

### 2.1. Plant Materials and Chemicals

*B. pendula* leaves were bought in herbalist office in market in Gornje Kolibe, Bosnia, and Herzegovina. The specimens were identified and the voucher is deposited in the Department of Pharmacognosy, Faculty of Pharmacy and Biochemistry, University of Zagreb, Zagreb, Croatia. Flavonoid and phenolic acid standards were purchased from Sigma-Aldrich (US). Their purity was 97% or higher. Methanol was of HPLC grade. Other reagents and chemicals were of analytical grade. Measurements were performed using Stat Fax 3200 (Awareness Technologies, USA) microplate reader and T70+ UV/Vis spectrometer (PG Instruments Ltd., GB).

### 2.2. Preparation of Extracts

Prior to the extraction, the dried leaves of* B. pendula* were milled and passed through a sieve of 850 *μ*m mesh size. Powdered plant material (2 g) was suspended with 20 mL of the appropriate solvent (80% ethanol or water) in a 50 mL Erlenmeyer flask. The extraction was performed in an ultrasonic bath (Bandelin SONOREX® Digital 10 P DK 156 BP, Germany) at ultrasonication power of 720 W and frequency of 35 Hz for 30 min at 80°C. The contents of the flasks were centrifuged (30 min at 3400 rpm). The supernatant was collected and evaporated at 30°C in rotavapor (ethanolic extract) or freeze-dried (aqueous extract).

### 2.3. Spectrophotometric Determinations of Total Phenols, Flavonoids, and Phenolic Acids

Total phenol (TP) content in the extracts was determined by the modified Folin-Ciocalteu colorimetric method [14], while the total flavonoid (TF) content was assessed by chelation of aluminum chloride [15]. Total phenolic acids (TPA) were determined using nitrite molybdate reagent [16]. For all the determinations, the modifications were used as described previously [4]. The contents of the analyzed substances in the extracts were expressed as mg/mL from calibration curves recorded for the standards and expressed as standard equivalents. Namely, TP, TF, and TPA were expressed as gallic acid, quercetin, and caffeic acid equivalents, respectively (Table 2).

### 2.4. HPLC Analysis of Phenolic Acids and Flavonoids

For determination of phenolic composition, phenolic acids and flavonoids were prepared in concentration of 0.2 mg/mL in methanol while the extracts were prepared in concentration of 2 mg/mL. For hydrolysis, in 1 mL of the corresponding extract solution 400 *μ*L 6 M HCl was added. The obtained mixtures were heated for 2 hours in water bath and then filtered to 5 mL volumetric flask. The precipitate on filter paper was washed with methanol and added to the flask contents to the volume. Phenolic acids and flavonoids were quantified using an HPLC instrument (Agilent 1200 series, Agilent Technologies, USA) equipped with an autosampler and DAD detector. Zorbax Eclipse XDB-C18 column (5 *μ*m, 12.5 mm × 4.6 mm, Agilent, USA) and Zorbax Eclipse XDB-C18 guard column were used for separation. Before the injections, the solutions of the standards and the extracts were filtered through a 0.45 *μ*m PTFE syringe filter. Mixture of water, methanol, and formic acid in proportions 93 : 5 : 2 (v : v : v) and 3 : 95 : 2 (v : v : v) were used as solvents A and B, respectively. Separation was performed at 40°C using following protocol: 0 min 20% B, 10 min 40% B, and 35 min 50% B. The flow rate was 1.0 mL/min. Applied volume was 10 *μ*L or 80 *μ*L for nonhydrolyzed or hydrolyzed samples, respectively. The peak assignment and identification were based on comparison of retention times of peaks in sample chromatogram and UV spectra with those of the standards. Components were quantified according to their respective standard calibration curve at 270 nm (rutin, myricetin, ellagic acid, and protocatechuic acid) or 290 nm (chlorogenic acid). Limit of detection (LOD) and limit of quantification (LOQ) were determined according to [17] (Table 1). The content of individual phenolic compounds is presented in (Table 3).

### 2.5. DPPH and ABTS Radical Scavenging Activity

DPPH radical scavenging activity (DPPH RSA) and ABTS radical scavenging capacity (ABTS RSA) were evaluated as described in [18] and [19], respectively. The reactions were performed at room temperature. To the free radical solution of appropriate concentration, extract solution was added. After incubation, absorbance was read at 545 nm or 734 nm for DPPH or ABTS free radical, respectively. RSA was calculated according to the equation RSA = (*A*_control_ − *A*_sample_)/*A*_control_ × 100, where *A*_control_ is the absorbance of the negative control (free solution without extract) and *A*_sample_ is the absorbance of the free radical solution containing extract. Concentration of the extract which scavenges 50% of of free radicals present in the solution (IC_50_ DPPH RSA and IC_50_ ABTS RSA) was calculated using regression analysis. BHA and Trolox were used as standard antioxidant for DPPH RSA and ABTS RSA, respectively.

### 2.6. Fe^2+^ Chelating Activity

Chelating activity (ChA) of the investigated substances toward ferrous ions was studied as described in [18]. To an aliquot of the methanolic extract solution (150 *μ*L), 0.25 mM FeCl_2_ solution (50 *μ*L) was added. After 5 min, the reaction was initiated by adding 1.0 mM ferrozine solution (100 *μ*L). Absorbance at 545 nm was recorded after 10 min of incubation at room temperature. A reaction mixture containing methanol (150 *μ*L) instead of extract solution served as a control. EDTA was used as the chelating standard. ChA was calculated using *A*_control_ (absorbance of the negative control, e.g., blank solution without test compound) and *A*_sample_ (absorbance of the substance solution). Using regression analysis, chelating activity was calculated as IC_50_ ChA, the concentration that chelates 50% of Fe^2+^ ions.

### 2.7. Total Antioxidant Activity

Total antioxidant activity (TAA) of extracts was determined using a spectrophotometer according to [20]. Briefly, an aliquot of 0.1 mL of sample solution was combined with 1 mL of reagent solution (0.6 M sulfuric acid, 28 mM sodium phosphate, and 4 mM ammonium molybdate). The tubes were capped and incubated in a thermal block at 95°C for 90 min. After the samples had cooled to room temperature, the absorbance was measured at 695 nm. Antioxidant activity was calculated based on the calibration curve of ascorbic acid and expressed as mg ascorbic acid equivalent (AAE) per g of dry weight.

### 2.8. Ferric-Reducing Antioxidant Power

Ferric-reducing antioxidant power (FRAP) was evaluated according to [21]. Fresh FRAP working solution was prepared by mixing 25 mL acetate buffer (300 mM), 2.5 mL of 2,4,6-tripyridyl-2-triazine solution (10 mM in 40 mM HCl), and 2.5 mL ferric chloride solution (20 mM). A mixture of 0.1 mL of extract solution was added to 0.9 mL of the FRAP solution and left in the dark at room temperature for 30 minutes. Absorbance was read spectrophotometrically at 593 nm. FRAP was calculated based on calibration curves of Trolox and expressed as mg Trolox equivalent (TE) per g of dry weight.

### 2.9. Determination of *α*-Glucosidase Inhibiting Activity

Inhibition of *α*-glucosidase was determined as reported earlier [22] with slight modification. In brief, 100 *μ*L of test samples dissolved in 10% DMSO (4, 2, 1, and 0.5 mg/mL solution) was incubated with 50 *μ*L of*α*-glucosidase from* Saccharomyces cerevisiae* Type I (Sigma-Aldrich, US) (1.0 U/mL dissolved in 0.1 M phosphate buffer, pH 6.8) for 10 min at 37°C. Afterwards, in reaction mixture, 50 *μ*L substrate (5 mM* p*-nitrophenyl-*α*-D-glucopyranoside prepared in the same buffer) was added and release of* p*-nitrophenol was measured at 405 nm spectrophotometrically after 5 min of incubation. Individual blanks for test samples were prepared to correct background absorbance where substrate was replaced with 50 *μ*L of buffer. Control sample contained 100 *μ*L 10% DMSO instead of test samples. Percentage of enzyme inhibition was calculated using equation AG = (*A*_control_ − *A*_sample_)/*A*_control_ × 100, where *A*_control_ is absorbance of the mixture without test compound (extract) and *A*_sample_ represents absorbance of samples containing extracts. As standard reference, acarbose was taken. Applying convenient regression analysis, IC_50_ (concentration of the test sample necessary to inhibit 50% activity of the enzyme) was obtained.

### 2.10. Alpha-Amylase Inhibition Assay

The assay was performed according to [23]. Extracts (25 *μ*L) at different concentrations and 25 *μ*L of 20 mM phosphate buffer (pH 6.9) containing porcine *α*-amylase (0.5 mg/mL) were preincubated at 25°C for 10 min. This was followed by addition of 25 *μ*L 0.5% soluble starch solution in the same buffer. The reaction mixture was incubated at 25°C for 10 min and then reaction was stopped with 50 *μ*L of 96 mM 3.5-dinitrosalicylic acid color reagent. Afterwards, the microplate was incubated in a boiling water bath for 5 min and cooled to room temperature. Absorbance was measured at 540 nm, and percent of enzyme inhibition was calculated as mentioned above. Control which represents 100% enzyme activity was prepared by replacing extract with 10% DMSO. Acarbose was used as a standard reference. The concentration of the extract needed to inhibit the activity of the enzyme by 50% (IC_50_) was calculated by regression analysis.

### 2.11. Cell Culture and Treatment

Human Caucasian hepatocyte carcinoma (Hep G2) cells from European Collection of Cell Cultures (ECACC) were maintained in an incubator at 37°C with a humidified atmosphere of 5% CO_2_ and cultured in MEM medium, supplemented with 10% (v/v) fetal bovine serum (FBS), 20 IU/mL penicillin, and 20 *μ*g/mL streptomycin. The medium was refreshed twice a week. For the experiments, cells were seeded into six-well plates. Plates were changed to FBS-free medium 24 h before the assay. For induction of hyperglycemic conditions, Hep G2 cells were cultured 24 h in MEM, supplemented with additional 20 mM glucose (positive control, D). For determining the influence of* B. pendula* extracts on GSH content in hyperglycemic conditions, Hep G2 cells were treated 24 h with 20 mM glucose plus either 0.5 mg/mL (D-0.5), 0.1 mg/mL (D-0.1), or 0.05 mg/mL (D-0.05) of* B. pendula* extracts. Negative control cells (C) were kept only in MEM medium which contained 5.56 mM glucose.

### 2.12. Reduced Glutathione Content (GSH)

Concentration of GSH was determined in hep G2 cell lysate that were treated with 20 mM glucose solution and different concentrations of extracts. GSH levels were quantified using a spectrophotometric assay, based on 2,2-dithiobisnitrobenzoic acid (DTNB or Ellman's reagent) at 37°C [24]. The production of a yellow colored 5-thio-2-nitrobenzoic acid was measured at 405 nm. Levels of GSH in treated cells were compared to negative and positive control cells.

### 2.13. Statistical Analysis

The experiments were performed in triplicate. The results were expressed as mean ± SD. Statistical comparisons were made using one-way ANOVA, followed by Dunnett's post hoc test and* t*-test for multiple comparisons with the control and between extracts, respectively. *P* values <0.05 were considered statistically significant. Statistical analyses were performed using the JMP version 6 from SAS software (SAS Institute, Cary, NC, USA).

## 3. Results and Discussion

### 3.1. Analysis of Total Phenols, Flavonoids, and Phenolic Acids

Chemical solvents interfere with different natural compounds yielding qualitatively and quantitatively different extracts. Those differences are inevitably reflected on antioxidant and other biological properties of the extracts [9]. Therefore, for the purpose of studying its antidiabetic activity,* B. pendula* leaf was extracted using the two most common and relatively nontoxic solvents: water and ethanol. The content of phenolic compounds in the prepared extracts is presented in Table 2. The amount of total phenols and flavonoids in ethanolic extract was approximately twofold higher than the amount of flavonoids in aqueous extracts. The amount of phenolic acids, on the other hand, was higher in the aqueous extracts. This is in line with higher lipophilicity of flavonoids in comparison to phenolic acids which makes them better soluble in relatively nonpolar solvent, ethanol.

**Figure 1:**
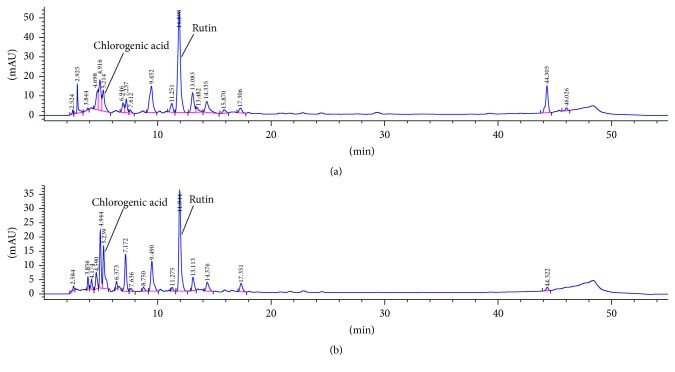
Chromatogram of ethanolic (a) and aqueous (b)* B. pendula* extract recorded at 320 nm.

The HPLC phytochemical analysis (Figure 1, Table 3) has confirmed that the main phenolic compound in* B. pendula *extracts is flavonoid rutin, while the other components are present in lower amounts. Similar to total flavonoids, the content of rutin was twofold higher in ethanolic than in aqueous extract. Among the phenolic acids, only chlorogenic acid was detected.

Previous studies have found that the flavonoids in* B. pendula* leaf are derivatives of myricetin, quercetin, kaempferol, apigenin, and luteolin. In those studies, quercetin derivatives were the most abundant while chlorogenic acid was present in the highest concentration along with* p*-coumaric acid derivatives [25]. In the extracts prepared in this study, however, analysis of UV spectra has shown that, besides rutin, other prominent peaks in the chromatogram (e.g., peaks at 9.49 min, 13.11, and 14.38 min) also belong to quercetin derivatives. This was confirmed by the analysis of the extracts subjected to acid hydrolysis. Both hydrolyzed extracts contained quercetin while a very low amount of myricetin was present only in hydrolyzed ethanolic extracts (the amount of myricetin was too low for quantification). In addition to flavonoids, protocatechuic acid, product of the flavonol degradation [26], was also detected, as well as low amount of ellagic acid. The presence of other used flavonoid aglycone and phenolic acid standards (baicalein, chrysin, hesperetin, luteolin, kaempferol, cinnamic, caffeic, chlorogenic, ferulic, rosmarinic, syringic, vanillic, and sinapic acid) was not detected in the extracts.

The three main proposed mechanisms through which antioxidants may play their protective role are hydrogen atom transfer, single electron transfer, and metal chelation [27]. The proportion of each of those mechanisms in total antioxidant activity of a herbal extract depends on various influences. Therefore, use of more than one method is recommended to give a comprehensive analysis of antioxidant efficiency of complex mixtures such as natural extracts. In the presented study, the following five assays were conducted: total antioxidant activity, DPPH and ABTS radical scavenging assay, and chelating and ferric-reducing antioxidant power assay. BHA, ascorbic acid, EDTA, and Trolox, antioxidants and ion chelators often employed in the food and pharmaceutical industry, were used as positive controls [28].

Comparison of antioxidant activities of the prepared extracts is presented in Table 4. Radical scavenging ability for DPPH free radical did not differ statistically between the two extracts, but ethanolic extract was better ABTS radical scavenger. However, as shown by markedly lower IC_50_ values, aqueous extracts were shown to be better Fe^3+^ ion chelator of metal ions than ethanolic extract. On the other hand, TAA and FRAP, methods that are based on reducing properties of the chemical species, were higher in case of ethanolic extract. Since phenolic compounds are considered to be the major compounds that contribute to the antioxidant activities of herbal extracts [29, 30], better antioxidant activity of ethanolic extract is not surprising.

**Figure 2:**
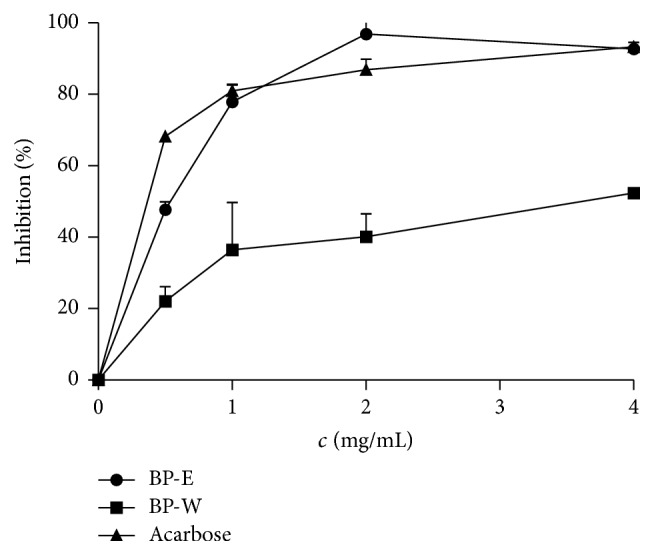
Alpha-glucosidase inhibitory activity of* B. pendula* extracts.

The prepared* B. pendula* leaf extracts were tested for their *α*-glucosidase and *α*-amylase inhibitory properties. While the extracts did not show any inhibitory activity toward *α*-amylase, their *α*-glucosidase activity was excellent and comparable to the activity of standard, antidiabetic drug acarbose (Figure 2). Ethanolic extract whose IC_50_ value did not statistically differ from acarbose was especially active (Table 4). In an attempt to determine the phytochemicals responsible for the observed *α*-glucosidase inhibitory activity, the activity of rutin and chlorogenic acid have also been tested. It was previously reported that chlorogenic acid may suppress postprandial hyperglycemia in rats by inhibiting *α*-glucosidase [31]. However, in the concentrations present in the active amounts of extracts in this study, rutin and chlorogenic acid did not present observable *α*-glucosidase inhibitory activity. If we compare the results obtained in this study with the IC_50_ values of rutin and caffeic acid needed for inhibition of *α*-glucosidase in previously published works [32, 33], we may observe that the concentration of those phenols in the present study may not be sufficient for displaying significant inhibitory potential. However, it has been found that rutin and chlorogenic acid display significantly lower anti-*α*-glucosidase activity then their nonconjugated counterparts, quercetin and caffeic acid, respectively [32, 33]. In addition, it seems that the level of glycosylation is inversely related to the inhibitory activity of quercetin derivatives [32]. It has been found that the combination of plant substances may have additive effect on *α*-glucosidase inhibition [34, 35]. Therefore, we may conclude that quercetin derivatives and other phenolic compounds that have been detected in the investigated extracts may play significant additive or even synergistic role in the observed *α*-glucosidase inhibitory activity. Besides phenolic substances,* B. pendula *leaf also contains triterpene compounds, betulin, betulinic acid, oleanolic acid, and lupeol [36]. It has been shown that oleanolic acid and related pentacyclic triterpenes may inhibit *α*-glucosidase* in vitro* in an uncompetitive and dose-dependent fashion with micromolar IC_50_ values [37]. Therefore, it is possible that these triterpenes significantly contribute to the observed inhibitory activity.

One of the consequences of hyperglycemia is elevated production of ROS which leads to the state of pronounced oxidative stress [1, 2]. Even though* B. pendula* extracts have previously been demonstrated to possess significant antioxidant potential in* in vitro *chemical models [38], to the best of our knowledge, birch leaf extracts have not been investigated in neither* in vivo* nor* in vitro* cellular models of diabetes. On the other hand, antioxidant and protective effects of rutin, which was the most prominent phenolic compound in the investigated extracts in our study, have been well investigated in* in vivo* models. For example, rutin can improve the antioxidant defense systems against iron overload-induced hepatic oxidative stress in rats. Such activity may be related to its antioxidant and metal chelation activities [39]. Furthermore, rutin was shown to possess neuroprotective and cardioprotective [40] effects in streptozotocin-induced diabetic models [41], as well as numerous other effects which may be beneficial in amelioration of diabetic complications [42]. Chlorogenic acid, another phenolic compound present in the investigated extracts, has well known antidiabetic properties which have been extensively reviewed [31] and linked to the observed diabetes protection of regular coffee consumption [43]. Some of its activity may be linked to antioxidant mechanisms since chlorogenic acid may ameliorate oxidative stress for renal injury in streptozotocin-induced diabetic nephropathy rats [44]. Therefore, we have aimed to investigate if* B. pendula* leaf extract can ameliorate consequences of hyperglycemia-induced oxidative stress in cellular model of diabetes.

**Figure 3:**
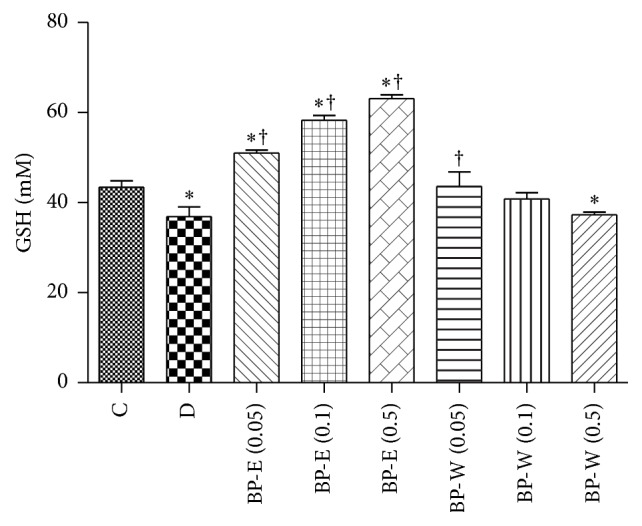
Glutathione (GSH) concentration in Hep G2 cells. C: cells in MEM; D: cells in MEM supplemented with 20 mM glucose; BP-E and BP-W: cells in MEM supplemented with 20 mM glucose and the corresponding* B. pendula* extract (numbers in bracket represent extract concentration in mg/mL); *∗* and †: value statistically different from C and D, respectively (*P* < 0.05, Dunnett's test). Values are average of 3 replications ± SD.

Oxidative stress in diabetes leads to decreased level of one of the most important antioxidants in the body, GSH [7, 45]. In order to evaluate the effect of* B. pendula* leaf extract on GSH concentration, Hep G2 cells were treated with high concentration of glucose. GSH was quantified using Cayman's GSH assay kit. The ability of aqueous and ethanolic extract to reduce oxidative stress in glucose-treated Hep G2 cells was investigated (Figure 3). Level of glutathione in glucose-treated cells (negative control) was significantly lower than in nontreated cells which served as confirmation that glucose has produced the oxidative stress. At the concentrations used in the experiment, both extracts reduced oxidative stress, as seen by significantly increased levels of glutathione in comparison with negative control. In addition to that, ethanolic extract was capable of increasing GSH concentration in comparison with normal control. This finding, along with the observed excellent activity in TAA, RP, and FRAP assay, the tests based on the reducing ability of the sample, might indicate that the electron-donating properties of ethanolic extract are mostly responsible for glutathione regeneration. Due to antioxidant properties of rutin and chlorogenic acid, which have been recorded in numerous studies [39, 44, 46], it may be concluded that a significant part of the observed activity could be attributed to the presence of those antioxidants.

## 4. Conclusions

*B. pendula* extracts posses significant antioxidant and antidiabetic properties as demonstrated by several antioxidant assays, ability to increase intracellular GSH concentration, and inhibition of *α*-glucosidase. Solvent choice can significantly affect biological properties of herbal extracts. In this study, ethanol was able to efficiently extract more or* B. pendula* leaf bioactive principles yielding the extract with higher content of phenolic antioxidants and better *α*-glucosidase inhibiting and GSH regenerating properties. Some of the observed biological properties could be attributed to rutin, natural flavonoid which was the main phenolic component of the investigated ethanolic extract. Future* in vitro* and* in vivo* studies are needed to further investigate antidiabetic potential of* B. pendula* ethanolic extract and its mechanism of action.

## Figures and Tables

**Table 1:** Calibration curve equation, limit of detection (LOD), and limit of quantification (LOQ) for the standards observed in chromatograms.

Standard	Calibration curve equation	*r* ^2^	LOD (*μ*g/mL)	LOQ (*μ*g/mL)
Chlorogenic acid	*y* = 2587.3*x* + 73.42	0.9996	0.036	0.110
Ellagic acid	*y* = 5194.5*x* + 101.67	0.9997	0.005	0.031
Myricetin	*y* = 2245.3*x* + 26.53	0.9999	0.112	0.340
Protocatechuic acid	*y* = 2633.3*x* + 17.58	0.9999	0.005	0.016
Quercetin	*y* = 2200.2*x* − 36.75	0.9998	0.027	0.083
Rutin	*y* = 1255.1*x* + 25.37	0.9998	0.013	0.039

*y*: area under curve (AUC, arbitrary units); *x*: concentration of the standard (*μ*g/mL).

**Table 2:** Total flavonoids (TF), phenolic acids (TPA), and phenols (TP) in *B. pendula *ethanolic (BP-E) and aqueous (BP-W) leaf extract.

Extract	TF (mg/g)	TPA (mg/g)	TP (mg/g)
BP-E	45.0 ± 2.8^A^	22.3 ± 0.5^A^	160.4 ± 10.6^A^
BP-W	20.1 ± 1.6^B^	35.9 ± 1.4^B^	80.6 ± 12.1^B^

^A-B^Differences within column (samples connected by the same capital letter are statistically different at *P* < 0.05).

**Table 3:** Results of HPLC analysis phenolic content in *B. pendula *ethanolic (BP-E) and aqueous (BP-W) leaf extract before and after hydrolysis.

Extract	Before hydrolysis (mg/g)		After hydrolysis		
	Rutin (mg/g)	Chlorogenic acid (mg/g)	Protocatechuic acid (mg/g)	Ellagic acid (mg/g)	Quercetin (mg/g)
BP-E	52.6	1.7	bLOQ	1.15	26.90
BP-W	25.8	1.8	28.55	2.25	23.45

bLOQ: below limit of quantification.

**Table 4:** Radical scavenging activity for DPPH (IC_50_ DPPH RSA) and ABTS (IC_50_ ABTS RSA) free radical, chelating activity (ChA), total antioxidant activity (TAA), ferric-reducing antioxidant power (FRAP), and *α*-glucosidase activity (IC_50_ AG) of *B. pendula *ethanolic (BP-E) and aqueous (BP-W) leaf extract.

Extract	IC_50_ DPPH RSA (*μ*g/mL)	IC_50_ ABTS RSA (*μ*g/mL)	IC_50_ ChA (*μ*g/mL)	TAA (mg AAE/g DW)	FRAP (mg TE/g DW)	IC_50_ AG (mg/mL)
BP-E	110.3 ± 14.3^A^	423.5 ± 35.8^A^	260.2 ± 26.5^A^	164.0 ± 2.9^A^	247.5 ± 3.4^A^	0.60 ± 0.03^A^
BP-W	87.5 ± 5.4^A^	563.3 ± 18.8^B^	118.4 ± 7.4^B^	142.9 ± 0.9^B^	150.9 ± 7.2^B^	2.88 ± 0.6^B^
Standard	^a^6.7 ± 0.9^B^	^b^42.9 ± 3.4^C^	^c^7.1 ± 0.4^C^	n.a.	n.a.	^d^0.50 ± 0.01^A^

^A–C^Differences within column (samples connected by the same capital letter are statistically different at *P* < 0.05).

AAE: ascorbic acid equivalents; TE: Trolox equivalents.

Standard: ^a^BHA; ^b^Trolox; ^c^EDTA; ^d^Acarbose.

n.a.: not applicable.
